# Bidirectional causal relationship between inflammatory cytokines and endometriosis at different sites

**DOI:** 10.1186/s12905-025-04058-7

**Published:** 2025-12-09

**Authors:** Shu-ping Huang, Ze-chao Zhang, Wen-jia Ding, Liang-ying Li, Wei-hong Li

**Affiliations:** 1 Guangxi University of Chinese Medicine, No. 10 Huadong Road, Xingning District, Nanning, 530000 PR China; 2 Ruikang Hospital Affiliated to Guangxi University of Chinese Medicine, Nanning, 530000 China

**Keywords:** Bidirectional Mendelian randomization, Endometriosis, Inflammatory cytokines

## Abstract

**Objective:**

This study aims to explore the genetic connection between inflammatory cytokines (IC) and endometriosis (EMs) to provide insights for the treatment of EMs.

**Methods:**

Data from two genome-wide association studies (GWAS) on IC and EMs were analyzed for single nucleotide polymorphisms (SNPs). Both forward and reverse Mendelian randomization (MR) analyses were conducted using inverse variance weighting. In the forward MR analysis, IC-related SNPs were used as instrumental variables with EMs as the outcome. Conversely, in the reverse MR analysis, SNPs associated with EMs were used as instrumental variables with IC as the outcome. Analyses were conducted using the TwoSampleMR package in R.

**Results:**

The forward MR analysis revealed no significant genetic correlation between 51 ICs and EMs (*P* > 0.05). The reverse MR analysis, however, revealed a significant genetic correlation between EMs and one type of IC (*P* < 0.05), with no significant correlations for the others (*P* > 0.05). Notably, specific inflammatory factors varied among different EMs sites (*P* < 0.05).

**Conclusion:**

Although ICs do not have a genetic influence on EMs, variations in inflammatory factors are observed in EMs patients, especially across different EMs sites. These findings suggest unique inflammatory responses at specific EMs sites.

**Supplementary Information:**

The online version contains supplementary material available at 10.1186/s12905-025-04058-7.

## Introduction

Endometriosis (EMs), characterized by pain and infertility, is a chronic inflammatory disease associated with significant disability in daily living, leading to socio-economic challenges and burdens [1]. EMs are one of the most common benign gynecologic growths in premenopausal women. It is estimated that 10 to 15% of women of reproductive age suffer from pelvic endometriosis, but its biological significance is unknown [2]. Despite its prevalence, the disease is still poorly understood. The present study proves that there is currently the lack of a reliable, noninvasive diagnostic test for the diagnosis of Ems [3]. Inflammatory cytokines (IC) are closely associated with the onset and progression of EMs and have the potential to be utilized as a diagnostic and therapeutic target for Ems [4]. However, the role of IC in EMs is still unclear. There are many reports indicating that IC plays a role in Ems [5, 6]. Are there specific associations between exposure and outcome? Is there a specific correlation between IC and EMs at different sites?

Given the aforementioned issues, Mendelian randomization (MR) offers a new analytical approach to elucidate the relationship between EMs and IC. MR uses single nucleotide polymorphisms (SNPs) as instrumental variables (IV) to infer the causality of observed associations between exposure or risk factors and clinically relevant outcomes. This approach has the advantage of minimizing confounding and reducing reverse causality bias [7, 8]. Due to the fruitful findings of large-scale genome-wide association studies (GWAS) conducted at IC and diseases, MR analysis has been widely used in a variety of scenarios. MR Has been used to study the relationship between EMs and IC in various parts of the body [4]. This has helped to clarify the specific mode of action between the two and has provided a new research direction for the diagnosis and treatment of EMs. In this study, a bidirectional MR design was used to investigate potential associations between IC and EMs.

## Materials and methods

### Study design description

**Fig. 1:**
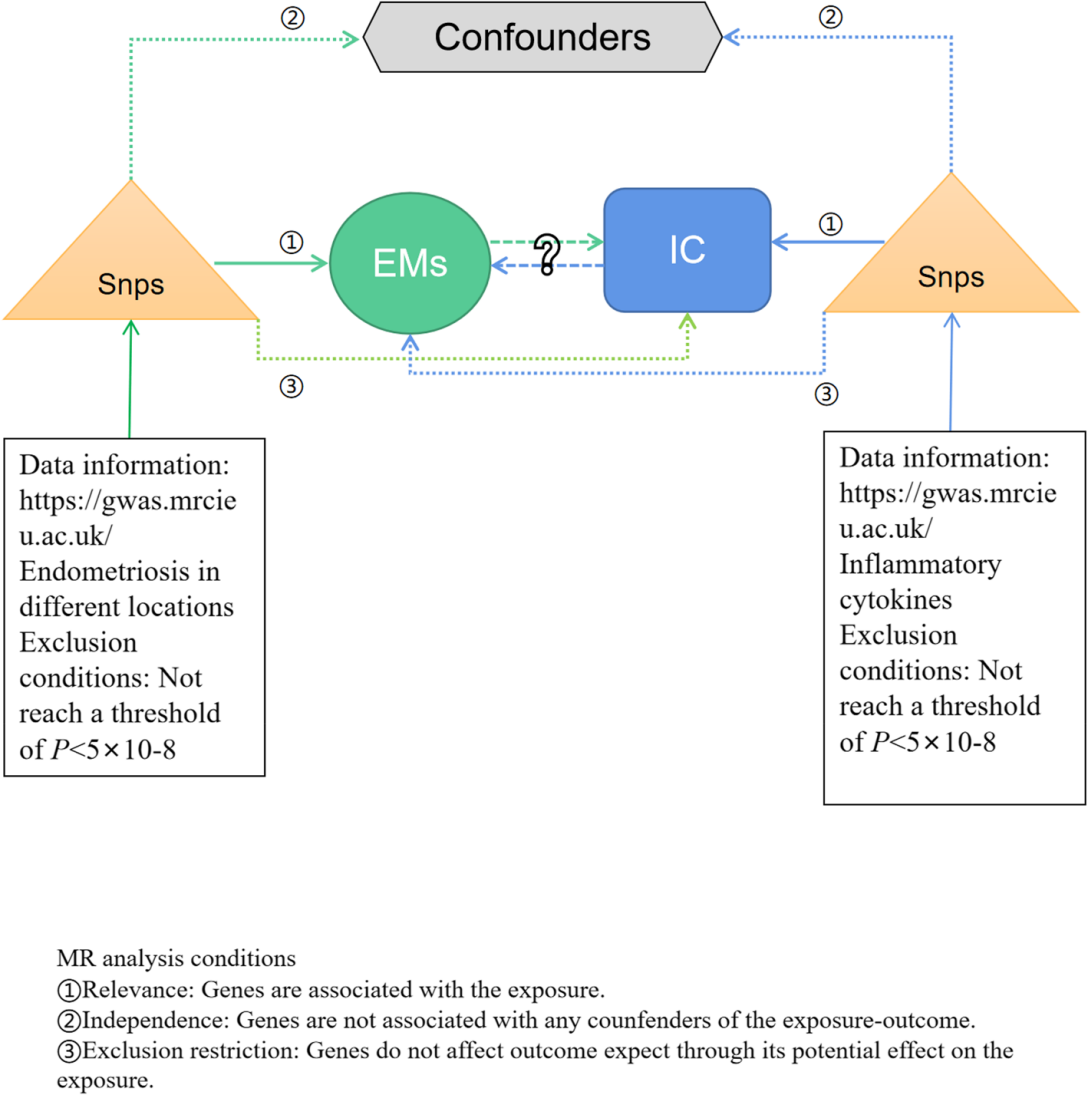
Flow chart of the bidirectional MR Study. The MR Analysis depends on three core assumptions: ①②③. Blue represents forward MR Analysis, IC is exposure, and EMs is outcome. Green represents reverse MR Analysis, EMs for exposure and IC for outcome. IC, inflammatory cytokines; EMs, endometriosis; MR, Mendelian randomization; SNP, single nucleotide polymorphism

The study design, depicted in Fig. 1, outlines the steps of this bidirectional Mendelian randomization (MR) study investigating the relationship between inflammatory cytokines (IC) and endometriosis (EMs). Summary statistics from GWAS for two MR analyses are used to investigate potential associations between IC and EMs. In the forward MR analysis, IC was considered as the exposure, with EMs as the outcome. Conversely, in the reversed MR, EMs was the exposure and IC was the outcome. The fundamental assumptions of MR are illustrated in Fig. 1. As this study relied on publicly accessible databases, it did not require ethical approval.

### MR instrumental variable selection

The instrumental variable (IV) for MR Analysis was derived from pooled results from two different GWAS. First, SNPs associated with exposure groups were screened using a genome-wide significance threshold (*P* < 5 × 10 − 8) [9]. Second, the independence of the selected SNPs was assessed based on pairwise linkage disequilibrium. When r2 > 0.001 (using a clustering window of 10000 kb), SNPs associated with more than one SNP and SNPs associated with higher *P* values were both deleted [10]. Linkage disequilibrium (LD) refers to the nonrandom association of alleles at different loci. Simply put, as long as two genes are not inherited completely independently, some degree of linkage is exhibited. r2: It is the correlation between 0 and 1, r2 = 1 indicates complete linkage disequilibrium between the two SNPs, while r2 = 0 indicates complete linkage equilibrium, meaning the assignment of the two SNPs is entirely random. kb: region length of linkage disequilibrium. r2 = 0.001, 10000 kb, indicating the removal of SNPs with an r2 greater than 0.001 within a 10000 kb range. Third, F-statistics were calculated to evaluate the strength of individual SNPs. SNPs were considered strong enough to mitigate the effects of potential bias when the F-statistic was greater than 10.

### Selection of EMs data sources and instrumental variables

The EMs data were derived from the MRC IEU UK Biobank GWAS pipeline version 2 (https://data.bris.ac.uk/data/dataset/pnoat8cxo0u52p6ynfaekeigi). All of this population data was based on EMs as the primary diagnosis and also included EMs at different sites. Endometriosis of uterus(EMsOU), Endometriosis of intestine(EMsOI), Endometriosis of ovary(EMsOO), Endometriosis of pelvic peritoneum(EMsOPP), Endometriosis of rectovaginal septum and vagina(EMsORSV), Endometriosis of fallopian tube(EMsOFT), Unspecified/other endometriosis(U-EMs). This GWAS was conducted to identify SNPs associated with EMs and EMs at various sites, which were chosen as IV (refer to Supplementary Table 1). All data in this study were obtained from the GWAS database, and there were no missing data.

### Selection of data source and instrumental variables of IC

IC data from the British Biological Bank (https://www.ebi.ac.uk/gwas/downloads/summary-statistics), which included 57,013 participants. 51 ICs were included in this GWAS. These 51 ICs were used for subsequent matching and analysis (refer to Supplementary Table 1).

### MR statistical analysis

SNPs for IC and EMs were used for subsequent forward and reverse MR analyses. The random effects inverse variance weighting (IVW) method, which relies on all core assumptions of MR, is a primary statistical approach used to estimate the potential bidirectional causality between EMs and IC [7]. When multiple IVs are available, IVW is the most effective analysis approach. This is because it takes into account variant specificity and causal estimation heterogeneity, while also conducting sensitivity analyses. These sensitivity analyses include the simple model, weighted model, weighted median, and MR-Egger regression method, which evaluate the robustness of study results [11]. However, IV influenced the results through other pathways, suggesting a potential horizontal pleiotropy effect, and indicating that the causal estimates of IVW may be biased. Therefore, MR-Egger was used to test for horizontal pleiotropy, and a *P* < 0.05 indicated the absence of horizontal pleiotropy. The MR heterogeneity test was used to determine the heterogeneity between SNPs. If there was heterogeneity, a random-effects model was used; otherwise, a fixed-effects model was used. To assess the impact of a single SNP on the results of the entire MR analysis, individual SNPs were excluded from the MR analysis one by one [12]. TwoSampleMR (version 0.5.6) in the R package (version 4.3.0) was used for the primary statistical analysis and charting [13]. The odds ratio (OR) and 95% confidence interval (CI) represent the extent of change in the resulting risk for each standard deviation increase in the exposure factor. Statistical significance was set at *P* < 0.05. All data in this study were obtained from the GWAS database, and there were no missing data.

## Results

### Forward MR

There were 51 IC included in this study including Interleukin-17(IL-17), Interleukin-8(IL-8), Interleukin-7(IL-7), Interleukin-4(IL-4), Eotaxin, CCL20, CCL23, CCL25, CCL28, CCL3, CCL4, CXCL1, CXCL10, CXCL11, CXCL5, CXCL6, CXCL9, Interleukin-6(IL-6), Interleukin-18(IL-18), Immunoglobulin E, Interleukin-11(IL-11), Interleukin-12(IL-12), Interleukin-23 (IL-23), Interleukin-13(IL-13), Interleukin-16(IL-16), Interleukin-17A(IL-17A), Interleukin-17C(IL-17C), Interleukin-17F(IL-17F), Interleukin-1 receptor antagonist protein, Interleukin-21(IL-21), Interleukin-25(IL-25), Interleukin-27(IL-27), Interleukin-2 receptor subunit alpha, Interleukin-31(IL-31), Interleukin-32(IL-32), Interleukin-34(IL-34), Interleukin-3(IL-3), Interleukin-36 alpha, Interleukin-36 beta, Interleukin-36 gamma, Interleukin-5(IL-5), Interleukin-6 receptor subunit alpha, Interleukin-9(IL-9), Toll-like receptor 4, Monocyte chemoattractant protein-1(MCP-1), Tumor Necrosis factor-Alpha(TNF-a), CRP, nerve growth factor(b-NGF), tumor Necrosis factor-beta(TNF-b), granulocyte colony-stimulating factor(G-CSF), Macrophage migration inhibitory factor(MIF). IVW results indicated that 51 IC were not significantly associated with EMs at the genetic level (*P* > 0.05) (see Supplementary Table 2). There was no significant horizontal pleiotropy among the SNPs (see Supplementary Table 2, *P* > 0.05). When combined with the IVW and MR-Egger methods, we found no significant heterogeneity associated with the association (refer to Supplementary Table 2, *P* > 0.05 for Cochran's Q). "There was no genetic correlation between EMs and IC exposure at the intestinal, uterine, vaginal, ovarian, or peritoneal pelvic sites (refer to Supplementary Table 2, *P* > 0.05).

### Reversed MR

**Fig. 2:**
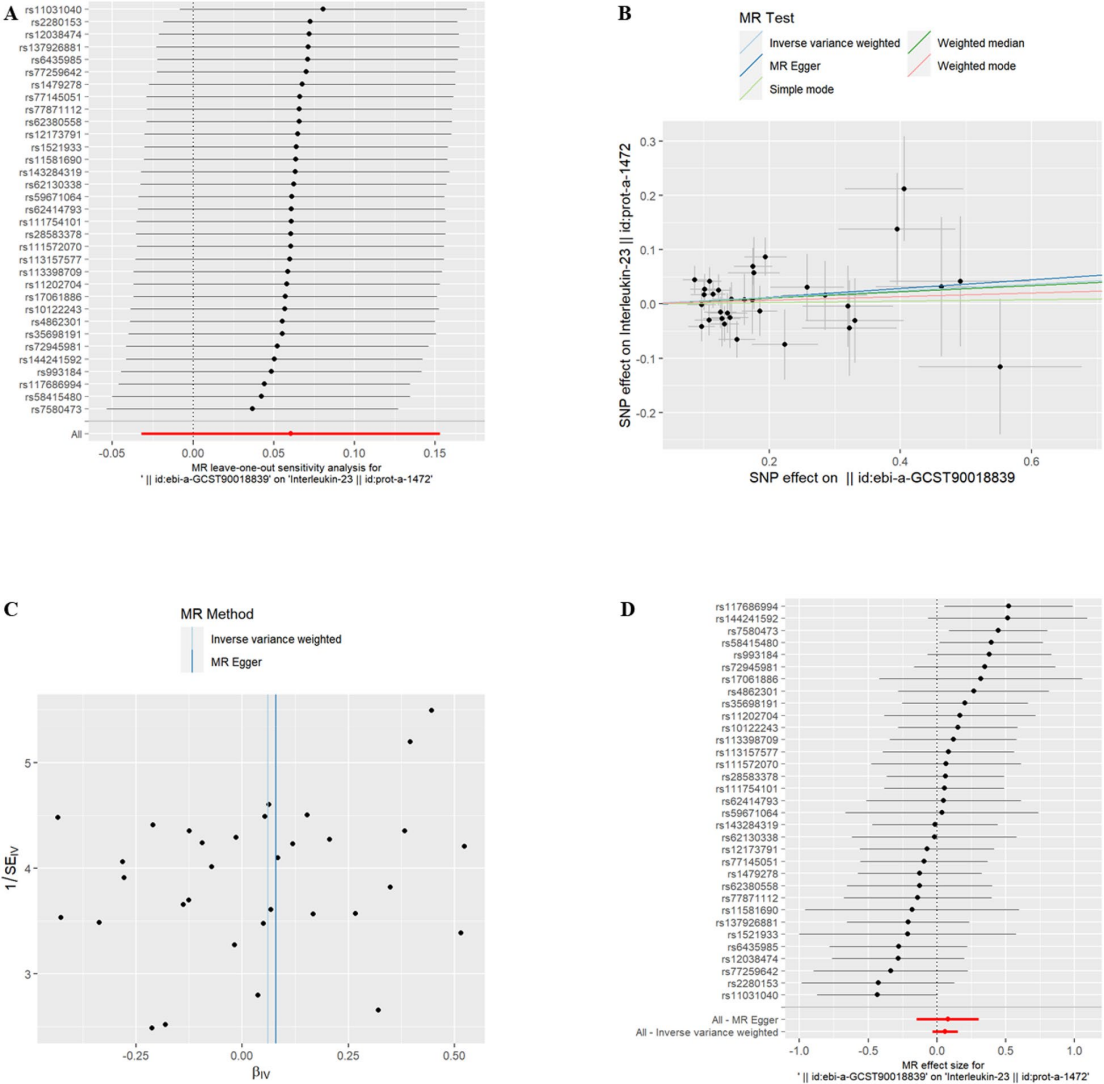
EMs and IC. **A** presents a forest plot of individual SNPs analyzed individually. Each horizontal solid line represents the estimated result using the Wald ratio method after excluding individual SNPs, designed to test the impact of a single SNP on the overall outcome. **B** displays a scatter plot in which each point represents an instrumental variable (IV). The line on each point represents the 95% confidence interval. The x-axis represents the effect of the SNP on the exposure, while the y-axis represents the effect of the SNP on the outcome. The colored lines illustrate the MR fitting results: light blue for Inverse Variance Weighted (IVW), dark blue for MR Egger, light green for Simple Mode, dark green for Weighted Median, and red for Weighted Mode. **C** features a funnel plot with the x-axis representing the IVW and MR values, and the y-axis indicating the instrumental variable (IV) values. The solid blue line corresponds to MR Egger, while the light blue line represents IVW. **D** displays a forest plot where each horizontal solid line represents the result estimated for a single SNP using the Wald ratio method. If the solid line lies entirely to the left of zero, the SNP is estimated to be associated with a decreased risk of the outcome. Conversely, if the solid line is entirely to the right of zero, the SNP is estimated to increase the risk of the outcome with increased exposure

1. EMs and IC The IVW results indicated that there was no significant correlation between EMs and 50 IC at the genetic level (*P* > 0.05). There was a significant correlation (*P* < 0.05) between EMs and 1 IC, Interleukin-23 (id: prot-a-1472) at the genetic level (refer to Table 1). The exclusion of the One-to-many forest plot did not indicate the presence of a single SNP that influenced the overall results, suggesting that the results of the MR analysis were supported by all included SNPs (Fig. 2A). From the combined results of the scatter plot and the forest plot, we can observe that the risk of Interleukin-23 outcome increased with greater EMs exposure (Fig. 2B, D). There was no significant horizontal pleiotropy observed among the SNPs (refer to Supplementary Table 2, *P* > 0.05). In addition, by combining Cochran's Q p-values in the IVW and MR-Egger methods (refer to Supplementary Table 2, *P* > 0.05), no significant association was found to be accompanied by heterogeneity (Fig. 2C).

**Table 1:** EMs IVW

id.exposure	id.outcome	pval	or	or_lci95	or_uci95
ebi-a-GCST90018839	prot-a-1472	0.024767241	1.248366302	1.0285633	1.515140999

**Fig. 3:**
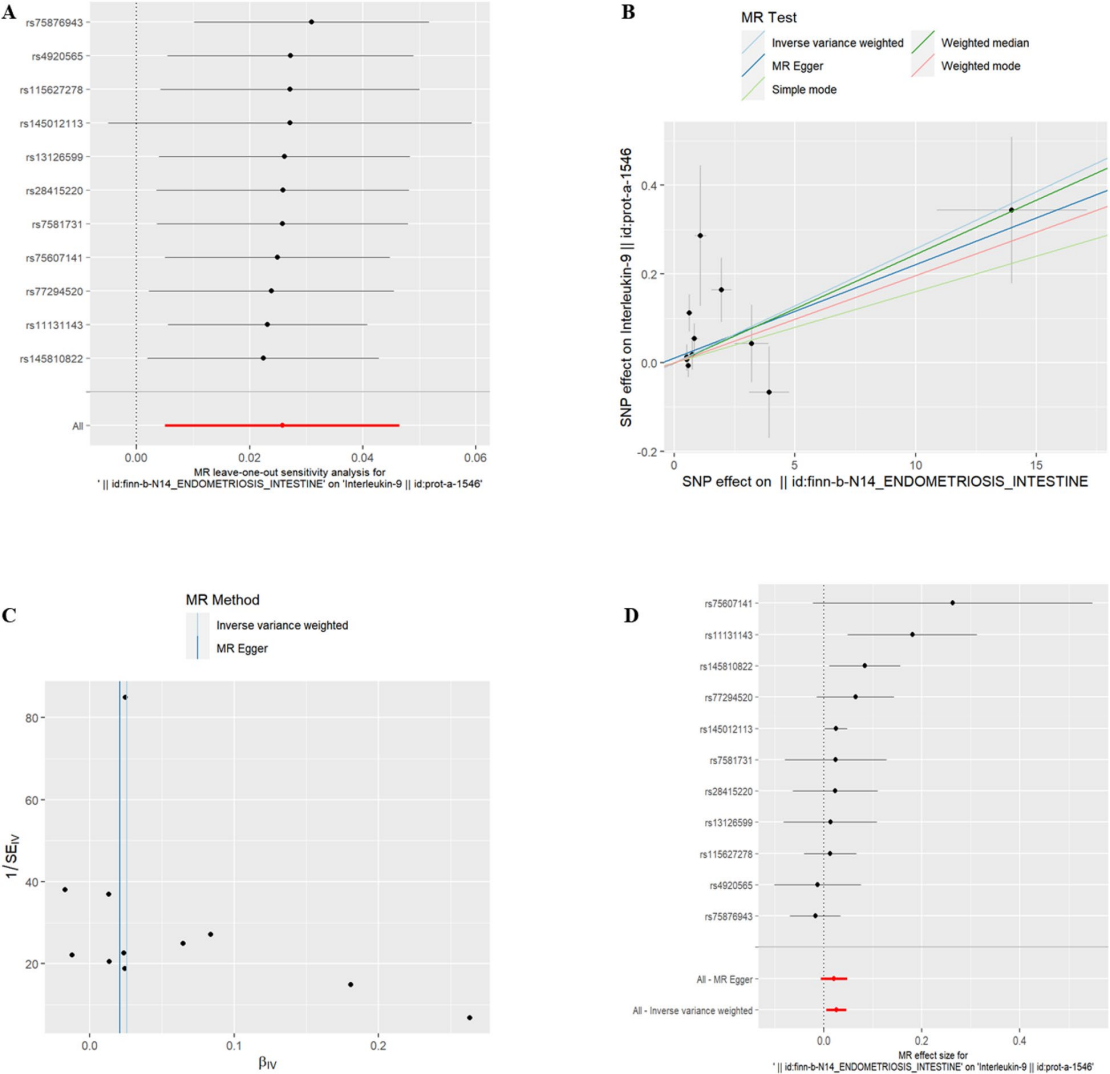
EMsOI and IL-9

**Fig. 4:**
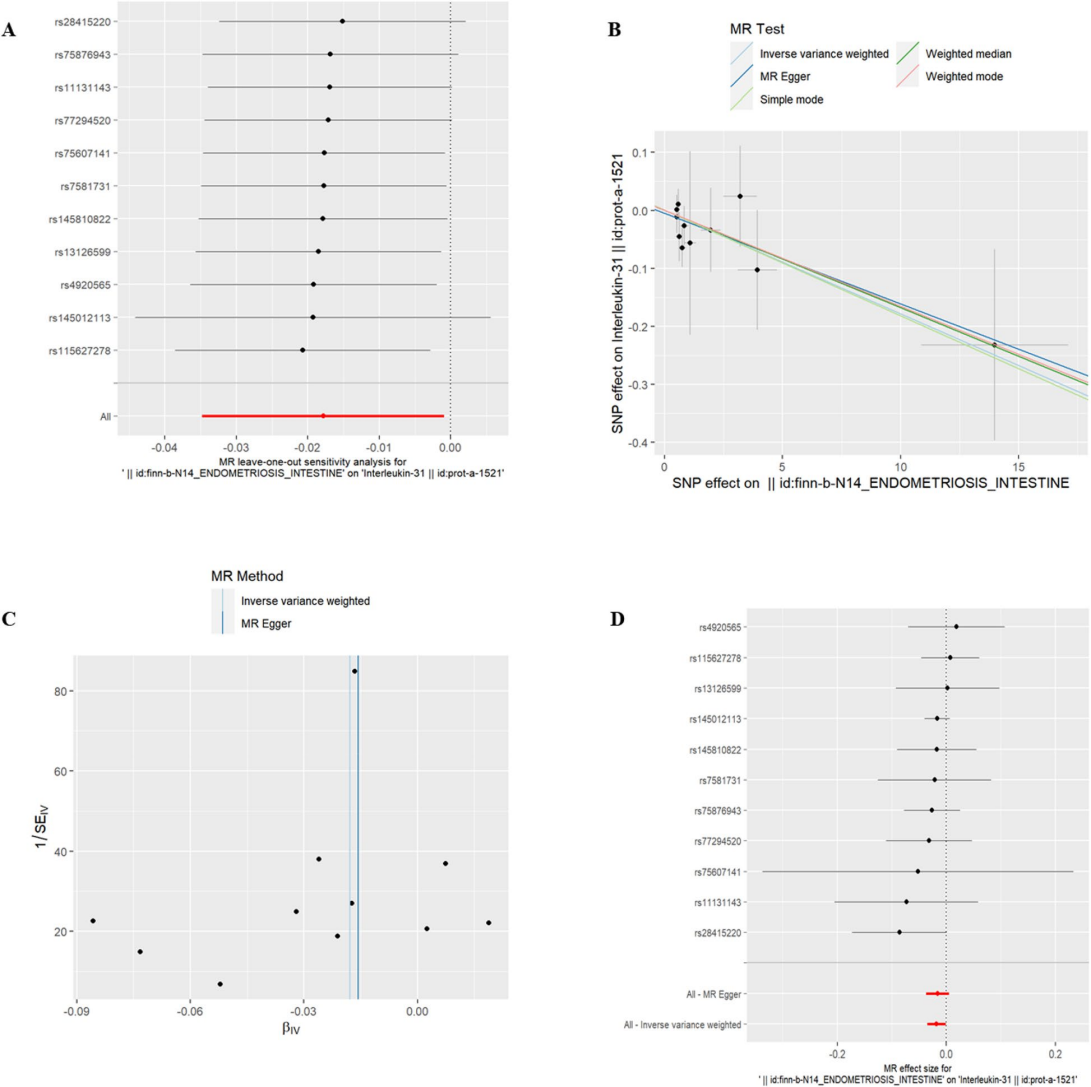
EMsOI and IL-31

**Fig. 5:**
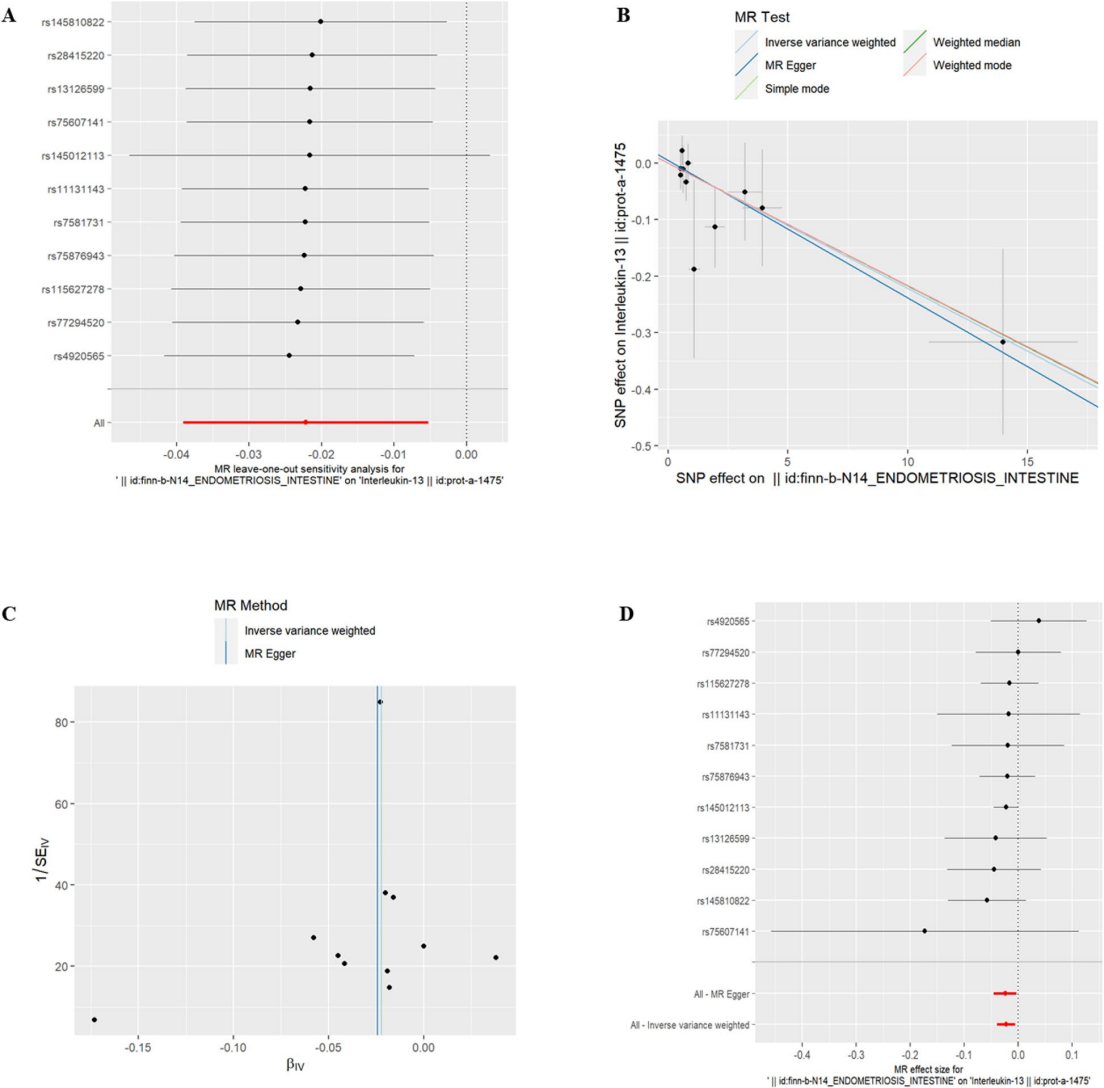
EMsOI and IL-13. **A** presents a forest plot of individual SNPs analyzed individually. Each horizontal solid line represents the estimated result using the Wald ratio method after excluding individual SNPs, designed to test the impact of a single SNP on the overall outcome. **B** displays a scatter plot in which each point represents an instrumental variable (IV). The line on each point represents the 95% confidence interval. The x-axis represents the effect of the SNP on the exposure, while the y-axis represents the effect of the SNP on the outcome. The colored lines illustrate the MR fitting results: light blue for Inverse Variance Weighted (IVW), dark blue for MR Egger, light green for Simple Mode, dark green for Weighted Median, and red for Weighted Mode. **C** features a funnel plot with the x-axis representing the IVW and MR values, and the y-axis indicating the instrumental variable (IV) values. The solid blue line corresponds to MR Egger, while the light blue line represents IVW. **D** displays a forest plot where each horizontal solid line represents the result estimated for a single SNP using the Wald ratio method. If the solid line lies entirely to the left of zero, the SNP is estimated to be associated with a decreased risk of the outcome. Conversely, if the solid line is entirely to the right of zero, the SNP is estimated to increase the risk of the outcome with increased exposure
(4) EMsOO and IC Interleukin-17C(id:prot-a-1483) and TNF-b(id:prot-c-4703_87_2) were found to have a genetic correlation with exposure to EMsOO (refer to Table 3, *P* < 0.05). The risk of Interleukin-17C and TNF-b increased with exposure to EMsOO. The exclusion of the One-to-many forest plot did not indicate the presence of a single SNP that influenced the overall results, suggesting that the results of the MR analysis were supported by all included SNPs (Figs. 6A and 7A). From the combined results of the scatter plot and the forest plot, we can observe that the risk of Interleukin-17C and TNF-b outcome increased with greater EMsOO exposure (Figs. 6B, D and 7B, D). In addition, by combining Cochran's Q p-values in the IVW and MR-Egger methods, no significant association was found to be accompanied by heterogeneity (Figs. 6C, and 7C).

(2) U-EMs and IC There was no significant association between U-EMs and 51 IC at the genetic level (refer to Supplementary Table 2, *P* > 0.05).
(3) EMsOI and IC Interleukin-13(id:prot-a-1475), Interleukin-9(id:prot-a-1546) and Interleukin-31(id:prot-a-1521) were found to have a genetic correlation with exposure to EMsOI (refer to Table 2, *P* < 0.05). Additionally, the risk of Interleukin-9 increased with EMsOI exposure. However, IL-13 and IL-31 were associated with a decreased risk. The exclusion of the One-to-many forest plot did not indicate the presence of a single SNP that influenced the overall results, suggesting that the results of the MR analysis were supported by all included SNPs (Figs. 3A, 4A, and 5A). From the combined results of the scatter plot and the forest plot, we can observe that the risk of Interleukin-9 outcome increased with greater EMsOIexposure (Fig. 3B, D). From the combined results of the scatter plot and the forest plot, we can observe that the risk of IL-13 and IL-31 outcome decreased with greater EMsOI exposure (Figs. 4B, D and 5B, D). In addition, by combining Cochran's Q p-values in the IVW and MR-Egger methods, no significant association was found to be accompanied by heterogeneity (Figs. 3C, 4C, and 5C).

**Table 2:** EMsOI IVW

id.exposure	id.outcome	pval	or	or_lci95	or_uci95
finn-b-N14_ENDOMETRIOSIS_INTESTINE	prot-a-1475	0.010233153	0.978071538	0.961658036	0.994765185
finn-b-N14_ENDOMETRIOSIS_INTESTINE	prot-a-1546	0.015000611	1.026089074	1.005014055	1.047606034
finn-b-N14_ENDOMETRIOSIS_INTESTINE	prot-a-1521	0.03894173	0.982325732	0.965837633	0.999095304

**Table 3:** EMsOO IVW

id.exposure	id.outcome	pval	or	or_lci95	or_uci95
finn-b-N14_ENDOMETRIOSIS_OVARY	prot-a-1483	0.004878733	1.078179725	1.023125123	1.136196828
finn-b-N14_ENDOMETRIOSIS_OVARY	prot-c-4703_87_2	0.00548799	1.715882656	1.17214382	2.511853271

**Fig. 6:**
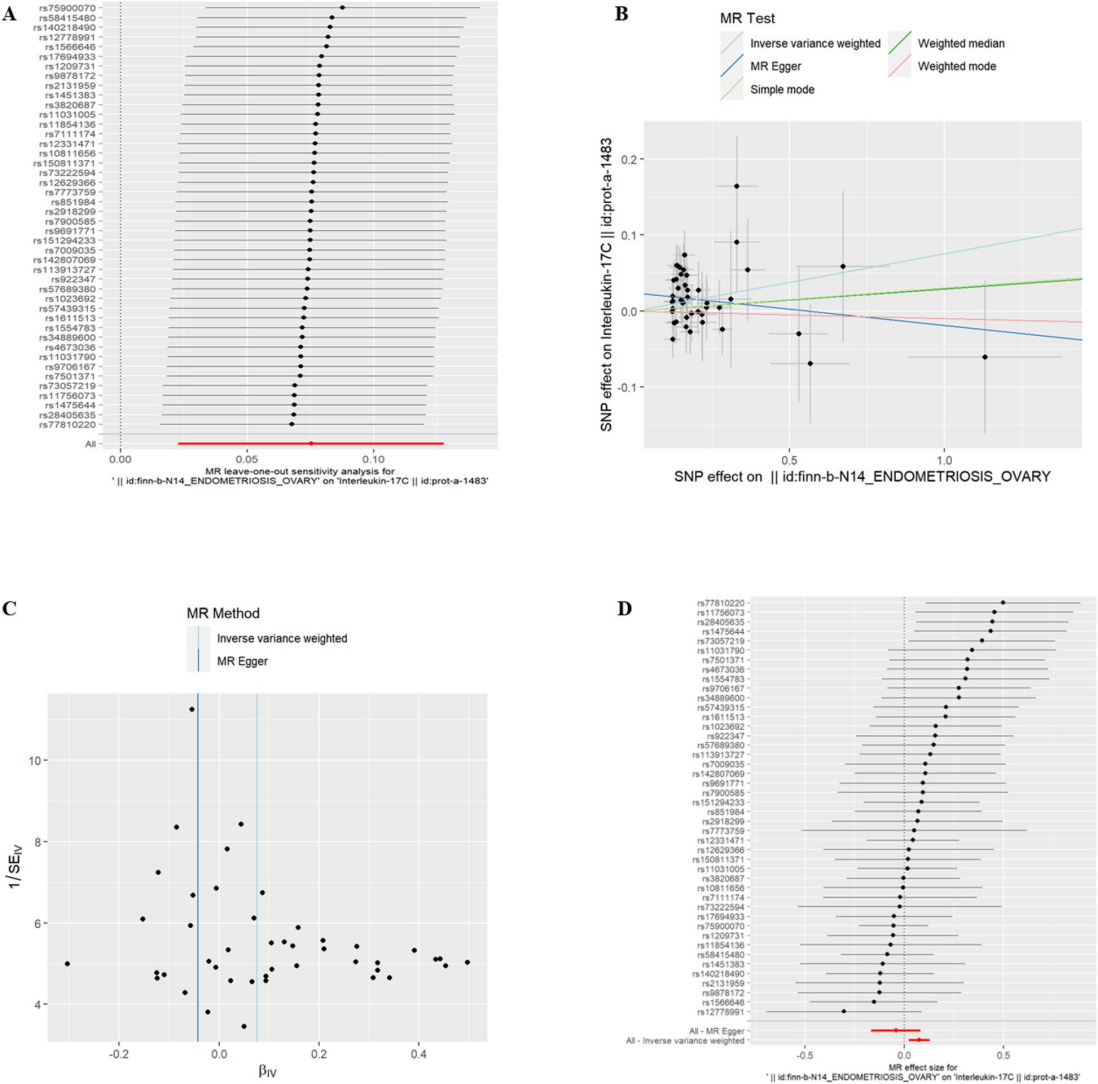
EMsOO and IL-17C

**Fig. 7:**
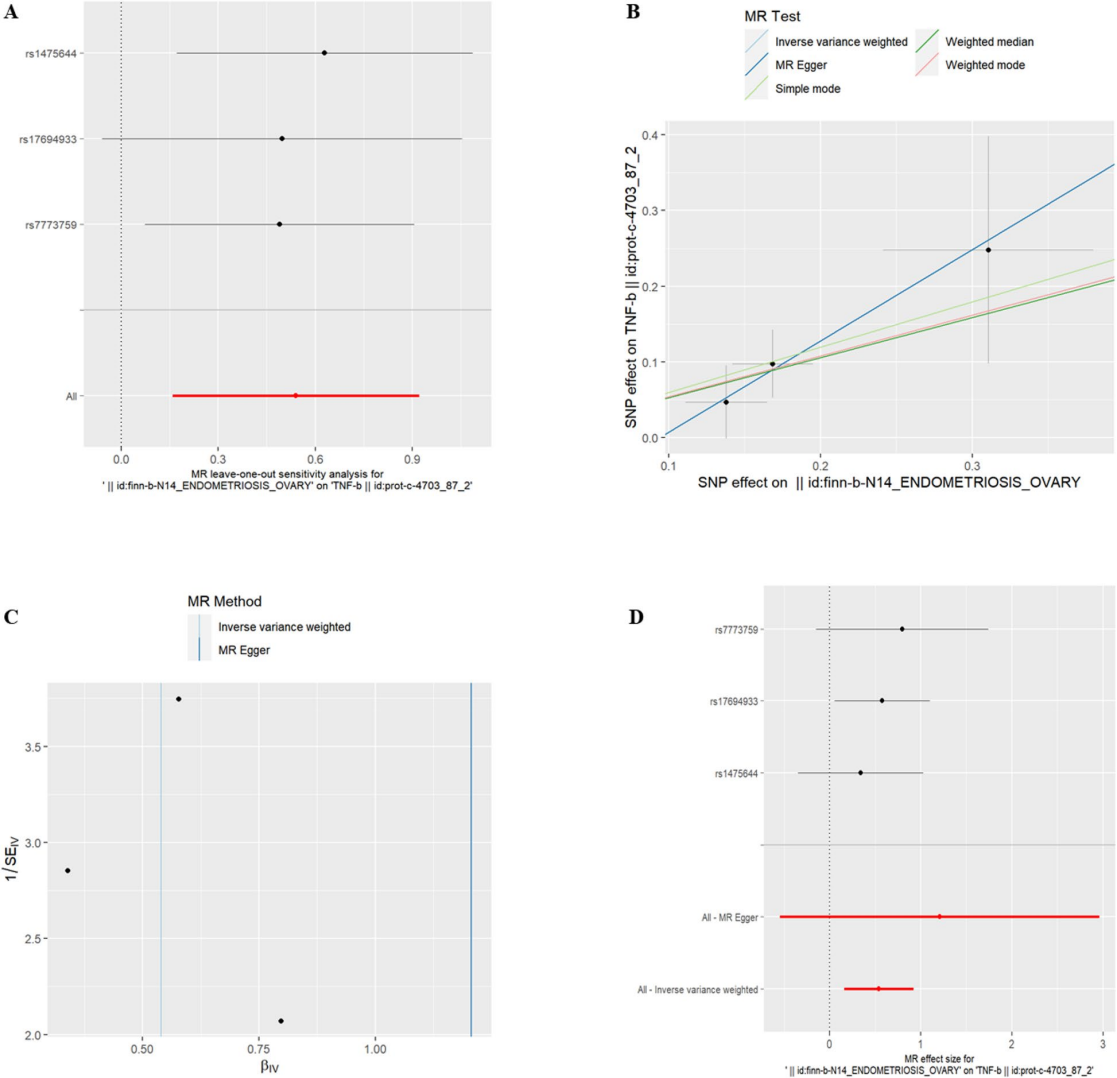
EMsOO and TNF-b. **A** presents a forest plot of individual SNPs analyzed individually. Each horizontal solid line represents the estimated result using the Wald ratio method after excluding individual SNPs, designed to test the impact of a single SNP on the overall outcome. **B** displays a scatter plot in which each point represents an instrumental variable (IV). The line on each point represents the 95% confidence interval. The x-axis represents the effect of the SNP on the exposure, while the y-axis represents the effect of the SNP on the outcome. The colored lines illustrate the MR fitting results: light blue for Inverse Variance Weighted (IVW), dark blue for MR Egger, light green for Simple Mode, dark green for Weighted Median, and red for Weighted Mode. **C** features a funnel plot with the x-axis representing the IVW and MR values, and the y-axis indicating the instrumental variable (IV) values. The solid blue line corresponds to MR Egger, while the light blue line represents IVW. **D** displays a forest plot where each horizontal solid line represents the result estimated for a single SNP using the Wald ratio method. If the solid line lies entirely to the left of zero, the SNP is estimated to be associated with a decreased risk of the outcome. Conversely, if the solid line is entirely to the right of zero, the SNP is estimated to increase the risk of the outcome with increased exposure

**Fig. 8:**
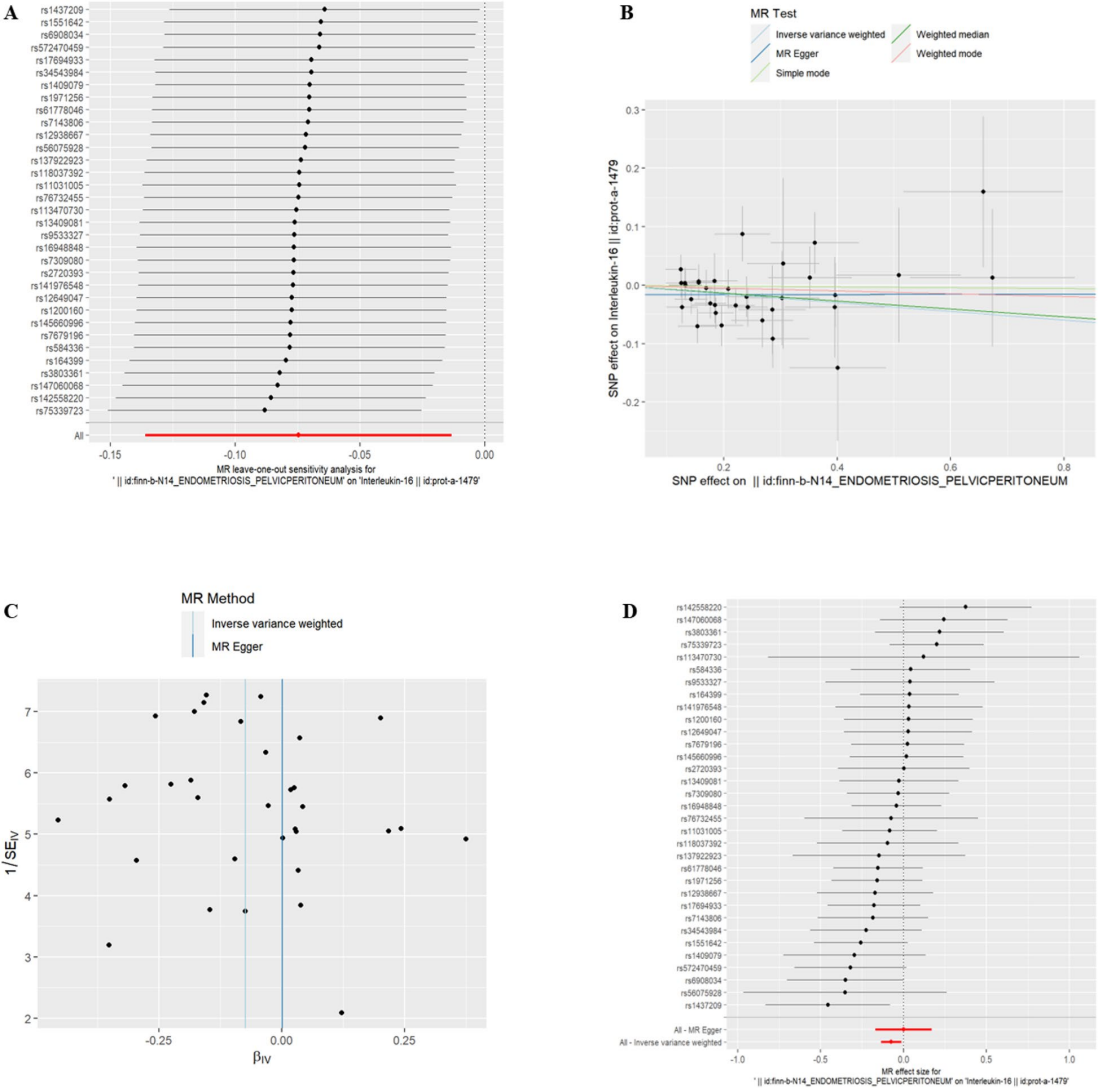
EMsOPP and IL-16

**Fig. 9:**
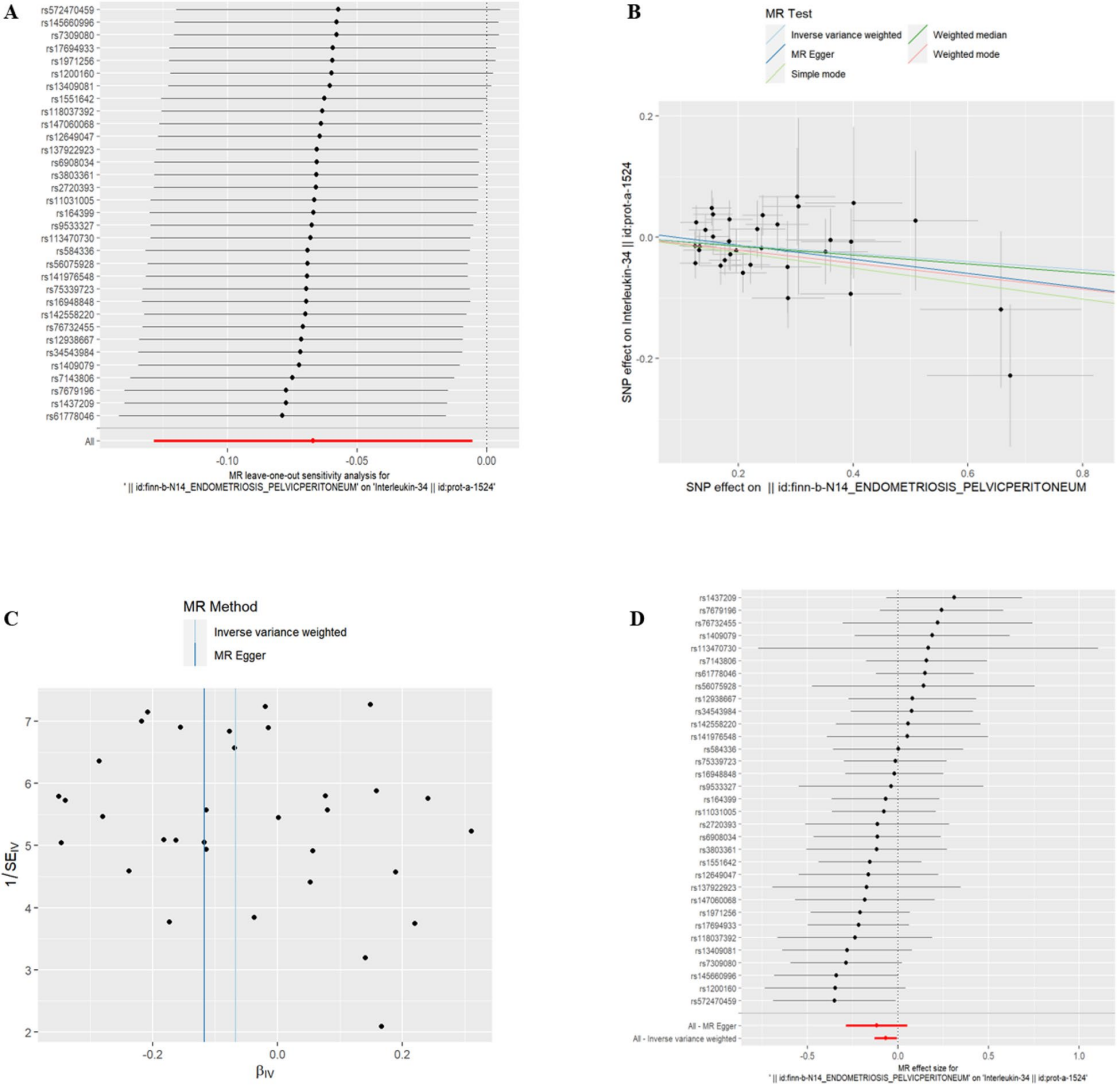
EMsOPP and IL-34

**Fig. 10:**
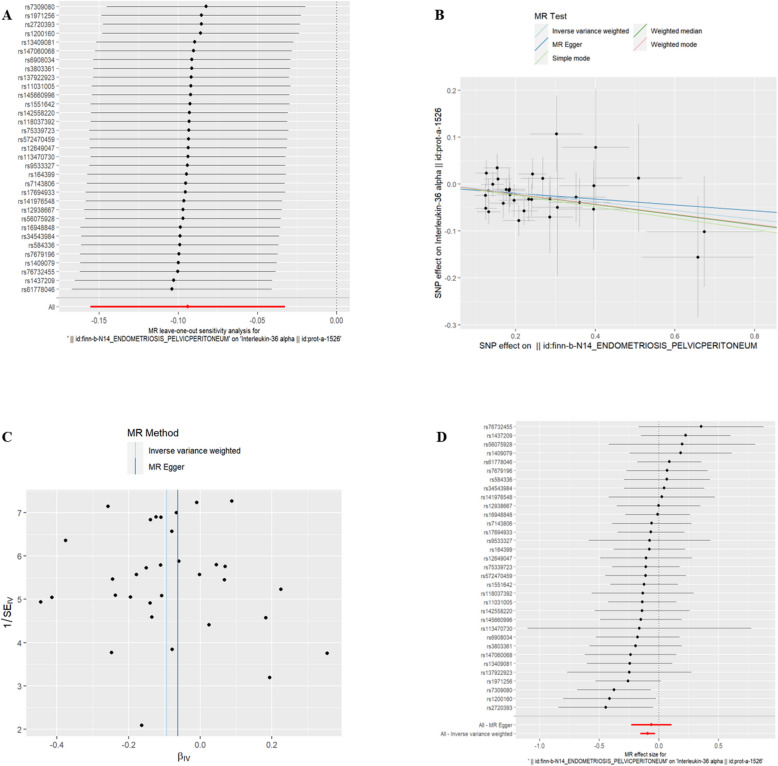
EMsOPP and IL-36 alpha. **A** presents a forest plot of individual SNPs analyzed individually. Each horizontal solid line represents the estimated result using the Wald ratio method after excluding individual SNPs, designed to test the impact of a single SNP on the overall outcome. **B** displays a scatter plot in which each point represents an instrumental variable (IV). The line on each point represents the 95% confidence interval. The x-axis represents the effect of the SNP on the exposure, while the y-axis represents the effect of the SNP on the outcome. The colored lines illustrate the MR fitting results: light blue for Inverse Variance Weighted (IVW), dark blue for MR Egger, light green for Simple Mode, dark green for Weighted Median, and red for Weighted Mode. **C** features a funnel plot with the x-axis representing the IVW and MR values, and the y-axis indicating the instrumental variable (IV) values. The solid blue line corresponds to MR Egger, while the light blue line represents IVW. **D** displays a forest plot where each horizontal solid line represents the result estimated for a single SNP using the Wald ratio method. If the solid line lies entirely to the left of zero, the SNP is estimated to be associated with a decreased risk of the outcome. Conversely, if the solid line is entirely to the right of zero, the SNP is estimated to increase the risk of the outcome with increased exposure

(5) EMsOPP and IC Interleukin-36 alpha(id:prot-a-1526), Interleukin-16(id:prot-a-1479) and Interleukin-34(id:prot-a-1524) were found to be associated with exposure to EMsOPP (refer to Table 4, *P* < 0.05). With exposure to EMsOPP, the risk of Interleukin-36 alpha, Interleukin-16, and Interleukin-34 decreased. The exclusion of the One-to-many forest plot did not indicate the presence of a single SNP that influenced the overall results, suggesting that the results of the MR analysis were supported by all included SNPs (Figs. 8A, 9A, and 10A). From the combined results of the scatter plot and the forest plot, we can observe that the risk of Interleukin-36 alpha, Interleukin-16, and Interleukin-34 outcome decreased with greater EMsOPP exposure (Figs. 8B, D, 9B, D and 10B, D). In addition, by combining Cochran's Q p-values in the IVW and MR-Egger methods, no significant association was found to be accompanied by heterogeneity (Figs. 8C, 9C, and 10C).

**Table 4:** EMsOPP IVW

id.exposure	id.outcome	pval	or	or_lci95	or_uci95
finn-b-N14_ENDOMETRIOSIS_PELVICPERITONEUM	prot-a-1526	0.002686653	0.910231053	0.85600691	0.967890048
finn-b-N14_ENDOMETRIOSIS_PELVICPERITONEUM	prot-a-1479	0.017044405	0.927992724	0.872732144	0.986752352
finn-b-N14_ENDOMETRIOSIS_PELVICPERITONEUM	prot-a-1524	0.032380977	0.935147404	0.879438438	0.994385314

**Fig. 11:**
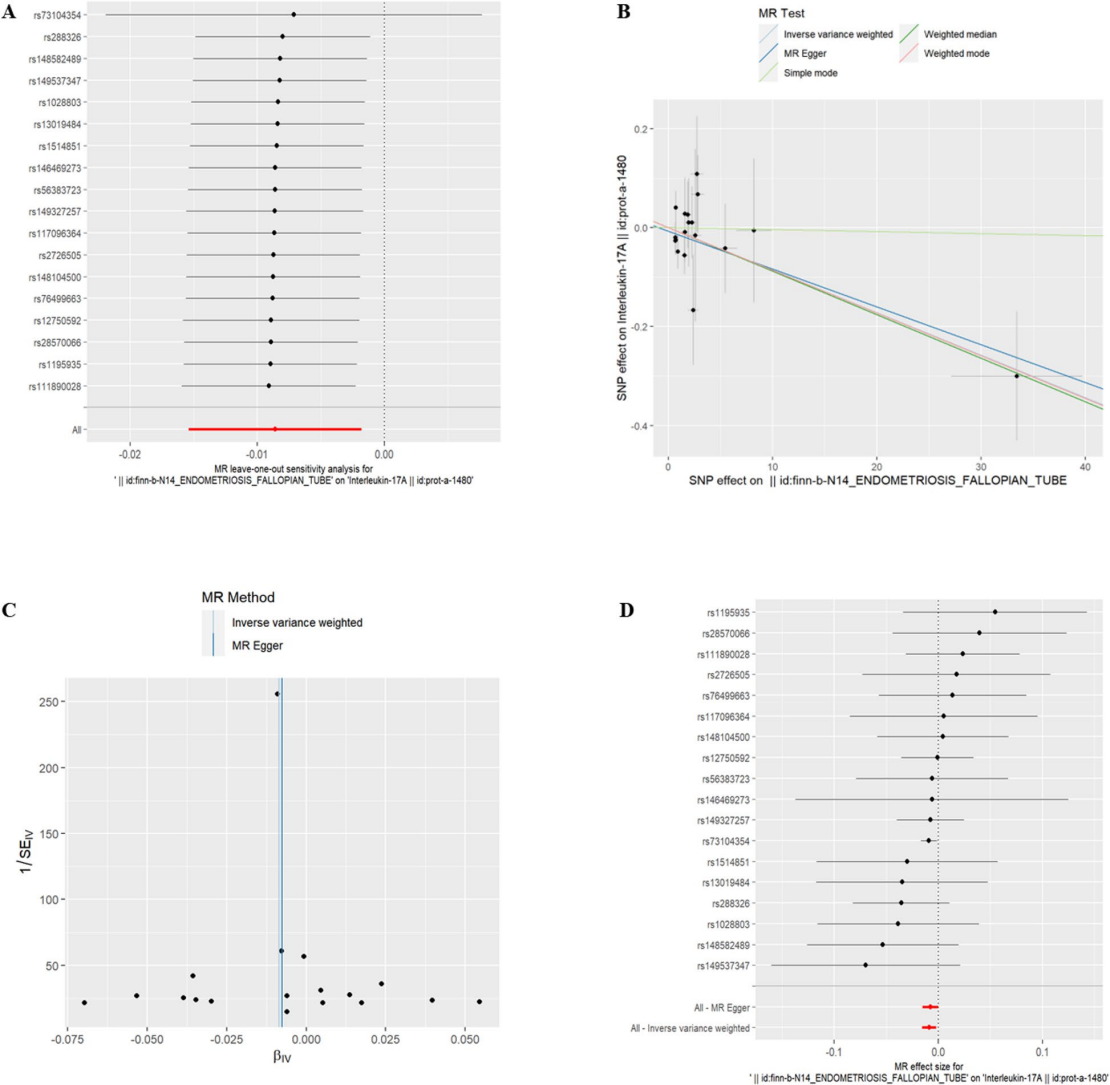
EMsOFT and IL-17A. **A** presents a forest plot of individual SNPs analyzed individually. Each horizontal solid line represents the estimated result using the Wald ratio method after excluding individual SNPs, designed to test the impact of a single SNP on the overall outcome. **B** displays a scatter plot in which each point represents an instrumental variable (IV). The line on each point represents the 95% confidence interval. The x-axis represents the effect of the SNP on the exposure, while the y-axis represents the effect of the SNP on the outcome. The colored lines illustrate the MR fitting results: light blue for Inverse Variance Weighted (IVW), dark blue for MR Egger, light green for Simple Mode, dark green for Weighted Median, and red for Weighted Mode. **C** features a funnel plot with the x-axis representing the IVW and MR values, and the y-axis indicating the instrumental variable (IV) values. The solid blue line corresponds to MR Egger, while the light blue line represents IVW. **D** displays a forest plot where each horizontal solid line represents the result estimated for a single SNP using the Wald ratio method. If the solid line lies entirely to the left of zero, the SNP is estimated to be associated with a decreased risk of the outcome. Conversely, if the solid line is entirely to the right of zero, the SNP is estimated to increase the risk of the outcome with increased exposure

(6) EMsOFT and IC There was a genetic correlation between Interleukin-17A(id:prot-a-1480) and EMsOFT exposure (refer to Table 5, *P* < 0.05). With EMsOFT exposure, the risk of IL-17A decreased. The exclusion of the One-to-many forest plot did not indicate the presence of a single SNP that influenced the overall results, suggesting that the results of the MR analysis were supported by all included SNPs (Fig. 11A). From the combined results of the scatter plot and the forest plot, we can observe that the risk of IL-17A outcome decreased with greater EMsOFT exposure (Fig. 11B, D). In addition, by combining Cochran's Q p-values in the IVW and MR-Egger methods, no significant association was found to be accompanied by heterogeneity (Fig. 11C).

**Table 5:** EMsOFT IVW

id.exposure	id.outcome	pval	or	or_lci95	or_uci95
finn-b-N14_ENDOMETRIOSIS_FALLOPIAN_TUBE	prot-a-1480	0.013391963	0.991445793	0.98471855	0.998218993

**Fig. 12:**
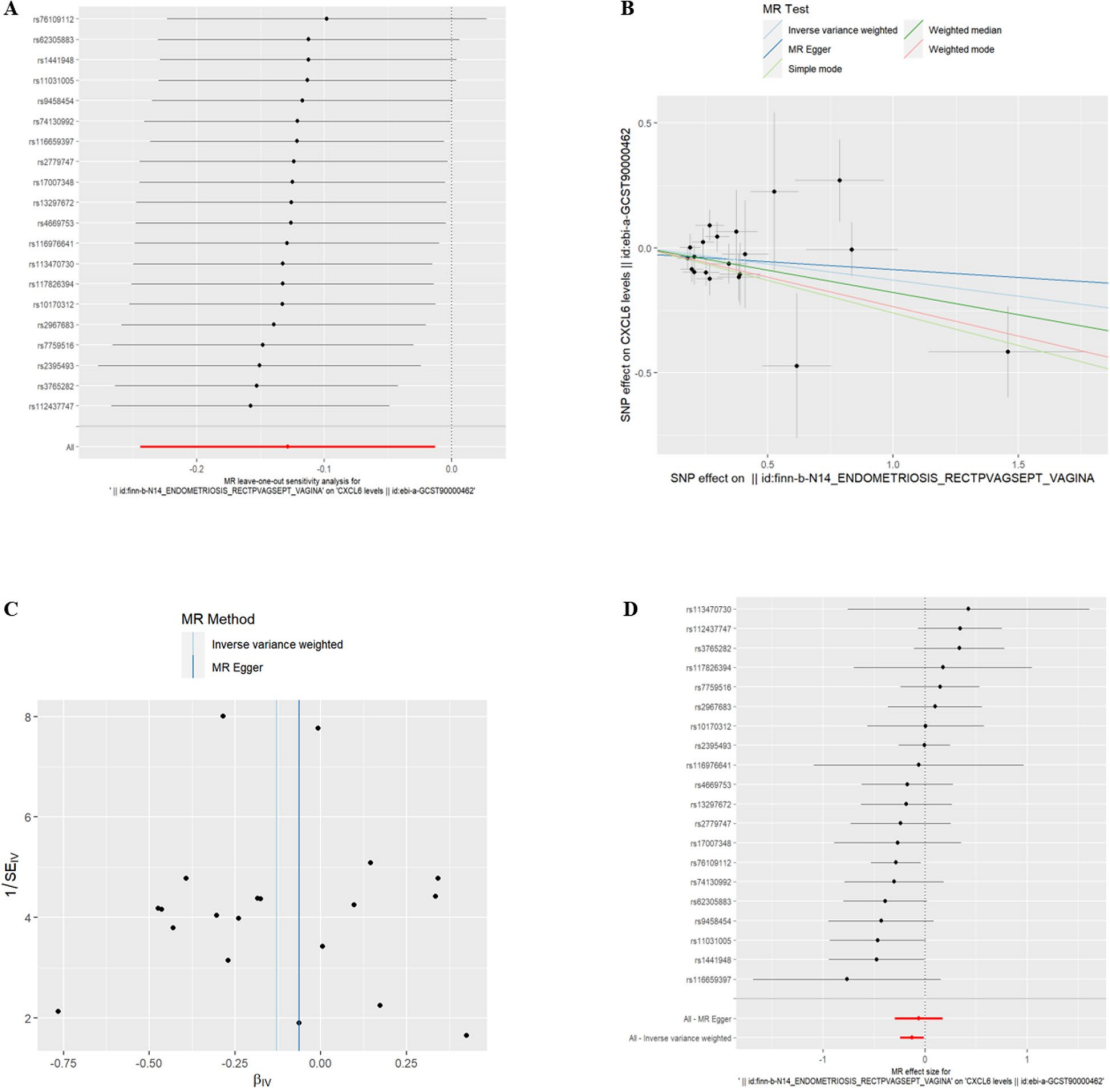
EMsORSV and CXCL6. **A** presents a forest plot of individual SNPs analyzed individually. Each horizontal solid line represents the estimated result using the Wald ratio method after excluding individual SNPs, designed to test the impact of a single SNP on the overall outcome. **B** displays a scatter plot in which each point represents an instrumental variable (IV). The line on each point represents the 95% confidence interval. The x-axis represents the effect of the SNP on the exposure, while the y-axis represents the effect of the SNP on the outcome. The colored lines illustrate the MR fitting results: light blue for Inverse Variance Weighted (IVW), dark blue for MR Egger, light green for Simple Mode, dark green for Weighted Median, and red for Weighted Mode. **C** features a funnel plot with the x-axis representing the IVW and MR values, and the y-axis indicating the instrumental variable (IV) values. The solid blue line corresponds to MR Egger, while the light blue line represents IVW. **D** displays a forest plot where each horizontal solid line represents the result estimated for a single SNP using the Wald ratio method. If the solid line lies entirely to the left of zero, the SNP is estimated to be associated with a decreased risk of the outcome. Conversely, if the solid line is entirely to the right of zero, the SNP is estimated to increase the risk of the outcome with increased exposure

(7) EMsORSV and IC There was a genetic correlation between CXCL6 levels(id:ebi-a-GCST90000462) and exposure to EMsORSV (refer to Table 6, *P* < 0.05). The risk of CXCL6 levels decreased with exposure to EMsORSV. The exclusion of the One-to-many forest plot did not indicate the presence of a single SNP that influenced the overall results, suggesting that the results of the MR analysis were supported by all included SNPs (Fig. 12A). From the combined results of the scatter plot and the forest plot, we can observe that the risk of CXCL6 outcome decreased with greater EMsORSV exposure (Fig. 12B, D). In addition, by combining Cochran's Q p-values in the IVW and MR-Egger methods, no significant association was found to be accompanied by heterogeneity (Fig. 12C).

**Table 6:** EMsORSV IVW

id.exposure	id.outcome	pval	or	or_lci95	or_uci95
finn-b-N14_ENDOMETRIOSIS_RECTPVAGSEPT_VAGINA	ebi-a-GCST90000462	0.029400622	0.879299749	0.78318978	0.987203954

**Fig. 13:**
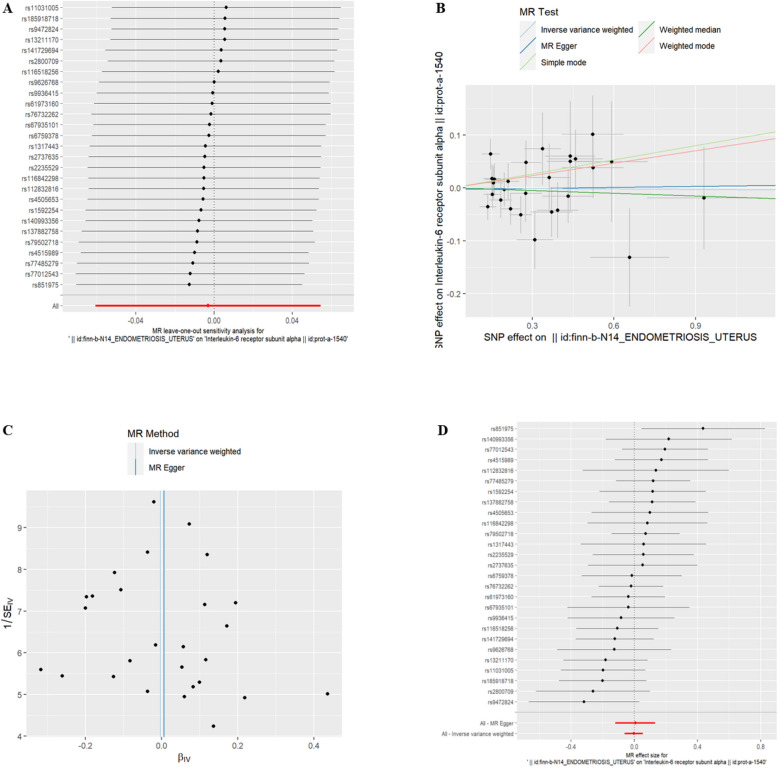
EMsOU and IL-6 receptor subunit alpha. **A** presents a forest plot of individual SNPs analyzed individually. Each horizontal solid line represents the estimated result using the Wald ratio method after excluding individual SNPs, designed to test the impact of a single SNP on the overall outcome. **B** displays a scatter plot in which each point represents an instrumental variable (IV). The line on each point represents the 95% confidence interval. The x-axis represents the effect of the SNP on the exposure, while the y-axis represents the effect of the SNP on the outcome. The colored lines illustrate the MR fitting results: light blue for Inverse Variance Weighted (IVW), dark blue for MR Egger, light green for Simple Mode, dark green for Weighted Median, and red for Weighted Mode. **C** features a funnel plot with the x-axis representing the IVW and MR values, and the y-axis indicating the instrumental variable (IV) values. The solid blue line corresponds to MR Egger, while the light blue line represents IVW. **D** displays a forest plot where each horizontal solid line represents the result estimated for a single SNP using the Wald ratio method. If the solid line lies entirely to the left of zero, the SNP is estimated to be associated with a decreased risk of the outcome. Conversely, if the solid line is entirely to the right of zero, the SNP is estimated to increase the risk of the outcome with increased exposure

(8) EMsOU and IC There was a genetic correlation between the exposure of the Interleukin-6 receptor subunit alpha (id:prot-a-1540) and EMsOU (refer to Table 7, *P* < 0.05). Exposure to EMsOU decreased the levels of Interleukin-6 receptor subunit alpha. The exclusion of the One-to-many forest plot did not indicate the presence of a single SNP that influenced the overall results, suggesting that the results of the MR analysis were supported by all included SNPs (Fig. 13A). From the combined results of the scatter plot and the forest plot, we can observe that the levels of Interleukin-6 receptor subunit alpha outcome decreased with greater EMsOU exposure (Fig. 13B, D). In addition, by combining Cochran's Q p-values in the IVW and MR-Egger methods, no significant association was found to be accompanied by heterogeneity (Fig. 13C).

**Table 7:** EMsOU IVW

id.exposure	id.outcome	pval	or	or_lci95	or_uci95
finn-b-N14_ENDOMETRIOSIS_UTERUS	prot-a-1540	0.049198689	0.827605172	0.685382872	0.999339709

## Discussion

In this bidirectional MR study, we identified a genetic link between EMs and IC. The forward MR analysis revealed no association between any of the 51 ICs and EMs, ruling out heterogeneity and pleiotropy. This suggests that these ICs do not exert a genetic influence on EMs. Conversely, the reverse MR analysis indicated that one IC, Interleukin-23, increased with EMs exposure, suggesting a differential presence in EMs patients.

We further investigated whether the genetic expression of ICs varied across different EMs sites. We observed an elevated levels associated with Interleukin-9 and a reduced levels with Interleukin-13 and Interleukin-31 in EMsOI. Interleukin-9, a potent proinflammatory cytokine, has been shown to exacerbate Ems [14, 15]. Both IL-13 and IL-31 are known inflammatory promoters [16, 17]. IL-13 levels were higher in patients with EMs than in normal women [18]. Our findings suggest that exposure to EMsOI increases the levels of the pro-inflammatory factor Interleukin-9, potentially worsening EMs, while reducing the levels of pro-inflammatory factors IL-13 and IL-31. The specific mechanisms behind these observations remain unclear and require further investigation. Exposure to EMsOO resulted in increased levels of Interleukin-17C and TNF-beta, both well-known pro-inflammatory factors. This suggests that exposure to EMsOO increases the levels of these proinflammatory cytokines, potentially exacerbating the severity of EMs. Interestingly, the levels of Interleukin-36 alpha, Interleukin-16, and Interleukin-34 decreased in EMsOPP. All three are known to induce and promote inflammation [19–23]. Exposure to EMsOFT was associated with a reduced levels of IL-17A, a key cytokine implicated in the pathophysiology of inflammatory and fibrotic diseases [24]. Elevated levels of IL-17A have been observed in the plasma of women with EMs and in lesions associated with Ems [25]. Similarly, exposure to EMsORSV was linked to decreased levels of CXCL6, a cytokine known to promote inflammation [26, 27]. Interestingly, exposure to EMsOU resulted in a decreased levels of Interleukin-6 receptor subunit alpha. IL-6 can bind to either the membrane-bound or soluble Interleukin-6 receptor subunit alpha, inducing anti-inflammatory classical signaling or proinflammatory trans-signaling, respectively [28]. The specific mechanism of action and dominant binding mode of the Interleukin-6 receptor subunit alpha in EMsOU warrant further investigation. The observed variations in genetic in EMsOPP, EMsOFT, and EMsORSV challenge the traditional belief that EMs is linked to heightened inflammation. The specific mechanisms that account for these discrepancies need further exploration. The reverse MR analysis of overall EMs did not show increased or decreased exposure to these proinflammatory cytokines, suggesting that these differences may be specific to certain sites. Previous studies have reported differences in clinical symptoms of EMs at different sites [29–31], which may be attributed to varying levels of inflammatory factors at these sites. This hypothesis requires experimental verification.

Our study findings showed that out of 51 ICs, only IL-23 exhibited differential expression in response to genetic, whereas the other 50 ICs did not show such variation. However, this pattern changed when specific EMs sites were examined. The levels of ICs fluctuated across different EMs sites, providing a basis for further research and potential diagnostic markers for site-specific EMs. These differences may explain the varying severity of EMs across sites and indicate that these key ICs could be potential therapeutic targets for site-specific treatment of EMs.The variation in IC levels across different EMs sites could have significant implications for prognosis and therapy. For example, EMsOI exhibited increased expression of IL-13 and IL-31, along with the pro-inflammatory factor IL-9. In contrast, EMsOO showed elevated levels of Interleukin-17C and TNF-beta. These differences may explain the varying severity of EMs across sites, suggesting that these key ICs could serve as potential therapeutic targets for site-specific treatment of EMs. Our study's strength lies in the use of Mendelian Randomization, which enables us to overcome issues of reverse causality and confounding bias that are frequently encountered in traditional observational studies. Furthermore, utilizing large-scale Genome-Wide Association Study GWAS) data for MR analysis improves the strength and accuracy of our findings.However, our study has limitations. Our findings suggest that the genetic of ICs varies across different EMs sites, the reasons for these discrepancies are not yet fully understood and warrant further investigation.

## Conclusion

In conclusion, our findings emphasize the intricate relationship between genetic factors and inflammatory processes in the pathogenesis of EMs. The findings highlight the potential of ICs as diagnostic markers and therapeutic targets for site-specific EMs, paving the way for future research in this area. Furthermore, our findings suggest a nuanced understanding of the role of inflammation in EMs. While the traditional belief suggests that EMs are linked to heightened inflammation, our findings suggest that this may not always be true. The genetic expression of certain proinflammatory cytokines, such as IL-17A, CXCL6, and Interleukin-6 receptor subunit alpha, decreased in specific EMs sites, contradicting the expected trend. This emphasizes the necessity for a more thorough and site-specific comprehension of the inflammatory processes involved in EMs. Furthermore, our study emphasizes the significance of taking into account the site-specific nature of EMs. The differential expression of ICs across various EMs sites suggests that the pathophysiology of EMs may vary depending on the site of occurrence. This has significant implications for the diagnosis and treatment of EMs, suggesting the necessity of site-specific therapeutic strategies. Despite these promising findings, our study has several limitations. The mechanisms that explain the observed associations between ICs and EMs have yet to be clarified. Future research should aim to uncover these mechanisms, which could offer valuable insights into the pathogenesis of EMs and guide the development of innovative therapeutic strategies. In summary, our study offers a new perspective on the role of inflammation in EMs, emphasizing the potential of ICs as diagnostic markers and therapeutic targets. However, further research is needed to fully understand the intricate relationship between genetic factors, inflammation, and EMs.

## Supplementary Information

Supplementary Material 1.

Supplementary Material 2.

Supplementary Material 3.

## Data Availability

No datasets were generated or analysed during the current study.
